# Use of lipid‐lowering therapies in patients with chronic kidney disease and atherosclerotic cardiovascular disease: 2‐year results from *G*etting to an impr*O*ved *U*nderstanding of *L*ow‐*D*ensity lipoprotein cholesterol and dyslipidemia management (*GOULD*)

**DOI:** 10.1002/clc.23923

**Published:** 2022-09-19

**Authors:** Aleesha Shaik, Mikhail Kosiborod, James A. de Lemos, Qi Gao, Katherine E. Mues, Shushama Alam, Deepak L. Bhatt, Christopher P. Cannon, Christie M. Ballantyne, Robert S. Rosenson, The GOULD Investigators

**Affiliations:** ^1^ The Cardiometabolic Disorders Unit Icahn School of Medicine at Mount Sinai New York New York USA; ^2^ Saint Luke's Mid America Heart Institute University of Missouri‐Kansas City Kansas City Missouri USA; ^3^ The George Institute for Global Health University of New South Wales Sydney Australia; ^4^ Division of Cardiology University of Texas Southwestern Medical Center Dallas Texas USA; ^5^ Amgen Inc. Thousand Oaks USA; ^6^ Brigham and Women's Hospital Heart and Vascular Center and Harvard Medical School Boston Massachusetts USA; ^7^ Baim Institute for Clinical Research Boston Massachusetts USA; ^8^ Department of Medicine Baylor College of Medicine Houston Texas USA

**Keywords:** atherosclerosis, cholesterol, chronic kidney disease, coronary artery disease, ezetimibe, lipids, statins

## Abstract

**Background:**

Chronic kidney disease (CKD) is a known risk factor of atherosclerotic cardiovascular disease (ASCVD). Per the 2018 American Heart Association/American College of Cardiology cholesterol guidelines, high‐risk ASCVD patients with CKD and low‐density lipoprotein cholesterol (LDL‐C) levels ≥ 70 mg/dL should take a high‐intensity statin with ezetimibe and/or a proprotein convertase subtilisin/kexin type 9 inhibitor (PCSK9i).

**Objective/Methods:**

We examined the changes in use of lipid lowering therapies (LLT) over two years in 3304 patients with ASCVD and CKD in the *G*etting to an impr*O*ved *U*nderstanding of *L*ow‐*D*ensity Lipoprotein Cholesterol and Dyslipidemia Management (*GOULD*) observational cohort study.

**Results:**

Of those with eGFR <60 ml/min/1.73 m^2^, 21.6% (171/791) had intensification of LLT while 10.4% (82/791) had de‐escalation of LLT. Notably, 61.6% (487/791) had no change in LLT regimen over 2 years. Statin use was 83.2% (785/944) at baseline and 80.1% (634/791) at 2 years. Statin/ezetimibe use increased from 2.9% (27/944) to 4.9% (39/791). Statin discontinuation at 2 years was greater with lower eGFR levels across all cohorts.

**Conclusion:**

Despite the recommendations of multiscociety guidelines, statin use, while high, is not ubiquitous and rates of high‐intensity statin and ezetimibe use remain low in patients with CKD. There remains a significant opportunity to optimize LLT and achieve atheroprotective cholesterol levels in the CKD population.

## 1 INTRODUCTION

Chronic kidney disease (CKD) is an independent risk factor for cardiovascular disease (CVD) and is therefore considered a risk enhancer for the use of lipid‐lowering therapies (LLT).^1^ In the United States, CKD affects about 15% of adults, or 37 million people.^2^ The prevalence of atherosclerotic CVD (ASCVD) rises with worsening kidney disease.^3^ Adults with end‐stage renal disease (ESRD) have 10–20 times higher risk of CVD than the general population and 50% of all deaths in patients with ESRD are due to CVD.^4^, ^5^

Dyslipidemia is present in even early stages of CKD and progresses as renal function worsens. Patients with CKD have elevated triglyceride levels, decreased high‐density lipoprotein cholesterol levels, and varying low‐density lipoprotein cholesterol (LDL‐C) levels.^6^ Advanced lipoprotein testing and vascular ultrasound examination for atheromatosis in patients without diabetes and with or without CKD (not on statins) in the Observatorio Nacional de Atherosclerosis en NEFrologia (NEFRONA) study demonstrated that CKD patients had a higher prevalence of atheromatosis and increased triglyceride‐rich LDL‐C particles, which was independently and positively associated with atheromatosis.^7^ The Chronic Renal Insufficiency Cohort (CRIC) study demonstrated that adults with CKD and ASCVD taking moderate‐ to high‐intensity statins still had high ASCVD event rates, suggesting that additional LLT may be beneficial in reducing ASCVD risk.^8^ Alirocumab, a proprotein convertase subtilisin/kexin type 9 inhibitor (PCSK9i), and icosapent ethyl were both reported to be associated with fewer cardiovascular events across a broad range of renal function categories in the ODYSSEY OUTCOMES and REDUCE‐IT RENAL studies respectively.^9^, ^10^

The 2013 Kidney Disease: Improving Global Outcomes (KDIGO) guidelines recommend a statin or statin/ezetimibe combination therapy to reduce ASCVD risk in patients ≥ 50 years of age with CKD who are not on dialysis, as well as for those aged 18–49 years with known ASCVD and non‐dialysis dependent CKD.^11^ The 2018 American Heart Association (AHA)/American College of Cardiology (ACC)/Multispecialty cholesterol guidelines recommend a high‐intensity statin with ezetimibe and/or PCSK9i for high‐risk ASCVD patients with CKD and LDL‐C levels ≥ 70 mg/dL.^12^ While evidence for LDL‐C lowering in the prevention of ASCVD in CKD patients primarily derives from post hoc analyses, subgroup analyses of multiple clinical trials support benefit.^13^ A meta‐analysis of 13 studies showed that statins reduced the risk of major cardiovascular events in Stage 3 CKD (pooled hazard ratio [HR] 0.72, 95% confidence interval [CI] 0.67–‐0.78) and stage 4 CKD (pooled HR 0.78, 95% CI 0.40–0.96) patients. There was no significant benefit in patients with Stage 5 CKD and those on dialysis, but overall, more patients with Stages 4 and 5 CKD need to be included in lipid lowering trials.^14^

The primary objective of the *GOULD* multicenter observational registry (NCT02993120) is to evaluate changes in use of evidence‐based lipid‐lowering therapies and, secondarily, trends in LDL‐C levels over time in patients with ASCVD in the United States. Patients were enrolled between 2017 and 2018 and followed prospectively for 2 years.^15^ Only 17.1% of all patients with ASCVD had intensification of LLT and two‐thirds of patients continued to have suboptimal LDL‐C levels after 2 years.^16^ This report describes the change in LLT usage and LDL‐C levels in patients with both ASCVD and CKD.

## 2 METHODS

The design of the GOULD study has previously been reported. Eligible patients were at least 18 years of age, on stable LLT for at least four weeks, and with established ASCVD.^15^ Established ASCVD was defined as having one of the following conditions: prior myocardial infarction, coronary artery disease, coronary or other arterial revascularization, ischemic stroke or transient ischemic attack, carotid artery stenosis, or peripheral artery disease. Exclusion criteria included current or planned future participation in interventional clinical study with an investigational medical device or drug treatment; life expectancy less than 12 months; current or planned future pregnancy, and breastfeeding.

In total, 5006 patients were enrolled from 119 centers across the United States between December 2016 and July 2018. Of these sites, 14.3% were hospital‐based and 85.7% were nonhospital based; 15.1% were in rural areas and 84.9% were in urban areas. The lead physician specialties were: 45.8% cardiology, 44.9% internal medicine, 0.9% nephrology, 2.5% endocrinology, and 5.9% other. Each site obtained institutional review board approval and all patients consented to the study.

Enrolled patients were divided into three cohorts: (1) currently receiving a PCSK9i (*n* = 554), (2) LDL‐C ≥ 100 mg/dL and not on PCSK9i (*n* = 1801), and (3) LDL‐C 70–99mg/dL and not on PCSK9i (*n* = 2651). Patients with available data on baseline estimated glomerular filtration rate (eGFR) were further categorized by kidney function. Seven patients received chronic dialysis. Upon enrollment, baseline data were collected and prospective data collection was completed through chart review every 6 months for 2 years.

## 3 RESULTS

At baseline, 3304 patients had a reported eGFR; 944 (28.6%) with an eGFR <60 ml/min/1.73 m^2^. At 2 years, 2870 patients with a baseline eGFR had completed 2 years of chart reviews: 61 (2.1%) had eGFR <30 ml/min/1.73 m^2^ (Stages 4–5 CKD), 730 (25.4%) had eGFR 30‐<60 ml/min/1.73 m^2^ (Stage 3 CKD), 1623 (56.5%) had eGFR 60‐<90 ml/min/1.73 m^2^ (Stage 2 CKD), and 456 (15.9%) had eGFR ≥ 90 ml/min/1.73 m^2^.

**Figure 1:**
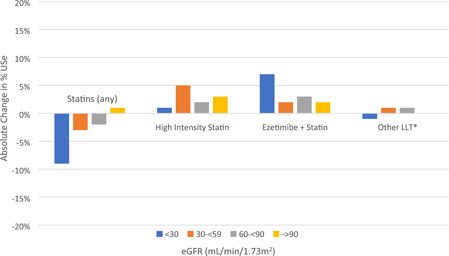
Change in lipid‐lowering therapy use over 2 years. Mild decreases in statin use were noted over two years across eGFR groups. There were modest increases in use of high‐intensity statin, particularly among eGFR 30–<60, and minimal change in the use of other lipid‐lowering therapies (LLT). While increases in ezetimibe/statin use were observed, overall prevalence remains low. *Other LLT include: Fibrate, Niacin, Mipomersen, Lomitapide, Cholestyramine, Colesevelam, Colestipol, and other unclassified medications.

Overall, the use of any statin remained relatively stable over 2 years (Figure 1). Among patients with a normal eGFR, statin use was 84.1% at baseline and 84.9% at 2 years. For patients with Stage 2 CKD, statin use was 84.8% at baseline and 83.4% at 2 years. Among patients with Stage 3 CKD, statin use was 82.8% and 80.3% at baseline and 2 years respectively. Finally, 87.5% of Stage 4–5 CKD patients were using statins at baseline compared with 78.7% at 2 years (Table 1).

**Table 1:** Use of lipid‐lowering therapies at baseline and 2 years by eGFR

	Baseline eGFR (ml/min/1.73 m^2^)							
	<30		30–<59		60–<90		≥ 90	
	Baseline (%)	2 Years (%)	Baseline (%)	2 Years (%)	Baseline (%)	2 Years (%)	Baseline (%)	2 Years (%)
Statins (any)	88	79	83	80	85	83	84	85
High‐intensity statin	40	41	39	44	41	43	43	46
Ezetimibe + Statin	1	8	3	5	4	7	4	6
Other LLT*	31	30	32	33	28	29	22	22
Fibrate	17	16	10	9	6	6	3	2
Fish oil	16	23	24	21	21	21	16	18

Table 1. While overall statin use is high in patients with chronic kidney disease and atherosclerotic cardiovascular disease, only modest increases in the use of high‐intensity statins, ezetimibe/statin, and other lipid‐lowering therapies (LLT) were observed over 2 years.

* Other LLT include: Fibrate, niacin, mipomersen, lomitapide, cholestyramine, colesevelam, colestipol, and other unclassified medications.

The use of high‐intensity statin increased from 40.3% (1328/3294) at baseline to 43.7% (1254/2870) at 2 years across all patients. Similarly, statin/ezetimibe use increased from 3.7% (122/3294) to 6.1% (176/2870). The percent change in LLT use over 2 years across all cohorts for each eGFR group is detailed in Figure 1. In the two LDL‐C cohorts, PCSK9i was added in 4.7% (117/2469) of patients.

Across all cohorts, 19.0% (546/2870) of patients had intensification of LLT while 9.8% (281/2870) had de‐escalation of LLT. Among the LDL‐C cohorts, 20.1% (496/2469) had intensification and 8.3% (205/2469) had de‐escalation of LLT (Tables 2 and 3). Notably, 64.8% (1860/2870) of patients had no change in their LLT regimen over 2 years. The greatest proportion with intensification was seen in the LDL‐C ≥100 mg/dL cohort at 26.1% (268/1028) with statin up‐titration being the largest contributor (Table 2). Statin discontinuation was a primary driver of de‐escalation in all cohorts (Table 3).

**Table 2:** Intensification of lipid‐lowering therapies over 2 years by cohort and eGFR

	PCSK9i cohort					LDL‐C 70–99 mg/dL cohort					LDL‐C ≥ 100 mg/dL cohort				
	Baseline eGFR (ml/min/1.73 m^2^)				Trend *p* value	Baseline eGFR (ml/min/1.73 m^2^)				Trend *p* value	Baseline eGFR (ml/min/1.73 m^2^)				Trend *p* value
	<30 (*n* = 7) (%)	30–<60 (*n* = 100) (%)	60–<90 (*n* = 234) (%)	≥ 90 (*n* = 60) (%)		<30 (*n* = 32)	30–<60 (*n* = 362) (%)	60–<90 (*n* = 830) (%)	≥ 90 (*n* = 217) (%)		<30 (*n* = 22) (%)	30–<60 (*n* = 268) (%)	60–<90 (*n* = 559) (%)	≥ 90 (*n* = 179) (%)	
**LLT Intensification**	0	15	13.7	5	*.236*	18.8	18.2	15.2	13.8	*.116*	27.3	29.1	25.6	22.9	*.152*
*Statin Added*	0	4	4.3	5	*.634*	0	3	1.8	2.8	*.925*	9.1	6	5.9	4.5	*.409*
*Statin Up‐titrated*	0	0	0.9	1.7	*.226*	12.5	9.4	6.3	5.1	*.014*	18.2	8.6	6.3	6.7	*.109*
*Ezetimibe Added*	0	3	5.1	0	*.701*	6.3	3.9	5.5	2.8	*.698*	13.6	9	7.7	6.1	*.165*
*PCSK9i Added*	0	3	1.7	0	*.243*	6.3	1.4	2.5	2.8	*.625*	0	9.7	7.9	7.3	*.715*
*Other LLT Added*	0	8	6.4	5	*.651*	6.3	4.1	3.3	4.1	*.637*	0	5.2	4.1	2.8	*.429*

*Note*: Intensification in lipid‐lowering therapies (LLT) was most common among the LDL‐C ≥ 100 mg/dL cohort and least among the PCSK9i cohort. Intensification was primarily driven by statin uptitration and the addition of ezetimibe. In the LDL‐C 70–99 mg/dL cohort, rates of statin uptitration were higher with worsening eGFR. Otherwise, there was no significant change in LLT use across eGFR groups among any cohort.

**Table 3:** De‐escalation of lipid‐lowering therapies over 2 years by cohort and eGFR

	PCSK9i cohort					LDL‐C 70–99 mg/dL cohort					LDL‐C ≥ 100 mg/dL cohort				
	Baseline eGFR (ml/min/1.73 m^2^)				Trend *p* value	Baseline eGFR (mL/min/1.73 m^2^)				Trend *p* value	Baseline eGFR (ml/min/1.73 m^2^)				Trend *p* value
	<30 (*n* = 7) (%)	30–<60 (*n* = 100) (%)	60–<9 (*n* = 234) (%)	≥ 90 (*n* = 60) (%)		<30 (*n* = 32) (%)	30–<60 (*n* = 362) (%)	60–<90 (*n* = 830) (%)	≥ 90 (*n* = 217) (%)		<30 (*n* = 22) (%)	30–<60 (*n* = 268) (%)	60–<90 (*n* = 559) (%)	≥ 90 (*n* = 179) (%)	
**LLT De‐escalation**	28.6	24	16.7	18.3	*.199*	9.4	6.4	8.4	7.4	*.641*	13.6	10.1	9.5	5.6	*.093*
*Statin Discontinued*	14.3	8	4.3	1.7	*.031*	9.4	3.3	3.3	3.2	*.409*	9.1	7.8	5.9	3.9	*.071*
*Statin Down‐titrated*	0	1	1.7	0	*.827*	6.3	0	2.7	3.7	*.024*	0	2.2	2.3	1.7	*.963*
*Ezetimibe Discontinued*	0	6	4.3	5	*.877*	3.1	0.8	0.8	0.9	*.65*	4.5	2.2	3.2	1.1	*.491*
*PCSK9i Discontinued*	14.3	8	6.8	13.3	*.508*	0	0	0	0	*n/a*	0	0	0	0	*n/a*
*Other LLT Discontinued*	0	7	6	1.7	*.321*	6.3	5.2	4.8	2.8	*.184*	4.5	5.6	4.5	3.9	*.43*

*Note*: De‐escalation in lipid‐lowering therapies (LLT) was most common among the PCSK9i cohort and least among the LDL‐C 70–99 mg/dL cohort. De‐escalation was primarily driven by statin discontinuation. In the PCSK9i cohort, rates of statin discontinuation were higher with worsening eGFR. In the LDL‐C 70–99 mg/dL cohort, rates of statin down‐titration were highest with eGFR <30. Otherwise, there was no significant change in LLT use across eGFR groups among any cohort.

Statin discontinuation at 2 years was greater with lower eGFR levels across all cohorts: 3.3% (15/46) of those with eGFR ≥ 90 ml/min/1.73 m^2^, 4.3% (70/1623) of those with eGFR 60–<90 ml/min/1.73 m^2^, 5.6% (41/730) of those with eGFR 30–<60 ml/min/1.73 m^2^, and 9.8% (6/61) of those with eGFR < 30 ml/min/1.73 m^2^. Muscle pain was indicated as the reason for statin discontinuation in 20.1% of patients across all cohorts, second to “other” (41.7%). Changing to another LLT (10.8%) and intolerance (7.8%) were other frequently identified reasons. Cost was a major driver of PCSK9i discontinuation (22.2%) across all cohorts, second to “other” (51.1%).

In the LDL‐C ≥ 100 mg/dL cohort, median LDL‐C decreased by 33.5 mg/dL to 94.5 mg/dL at 2 years in patients with eGFR of <30 ml/min/1.73 m^2^ and by 30 mg/dL to 91 mg/dL in those with eGFR 30–<60 ml/min/1.73 m^2^. The LDL‐C 70–99 mg/dL cohort demonstrated a similar trend: decrease by 10 mg/dL–71 mg/dL in patients with eGFR of <30 ml/min/1.73 m^2^ and by 4 mg/dL–77 mg/dL in those with eGFR 30–<60 ml/min/1.73 m^2^. In the PCSK9i cohort, median LDL‐C decreased by 33.5 mg/dL–57.5 mg/dL in patients with eGFR of <30 ml/min/1.73 m^2^ and remained stable in those with eGFR 30–<60 ml/min/1.73 m^2^, from 65.5 mg/dL–66 mg/dL. Overall, 34.0% (856/2518) of the study population achieved LDL‐C < 70 mg/dL by 2 years, 53.3% (204/383) of the PCSK9i cohort compared with 30.5% (652/2135) of the LDL‐C cohorts.

In the LDL‐C cohorts at baseline, the median non‐HDL‐C levels were 132.4 mg/dL, 126.0 mg/dL, 123 mg/dL, and 127 mg/dL in the eGFR <30, 30–<60, 60–<90, and ≥90 ml/min/1.73 m^2^ groups respectively. Remnant cholesterol levels, which were estimated by subtracting the sum of HDL‐C and LDL‐C from total cholesterol levels, were 39.4 mg/dL, 33 mg/dL, 30 mg/dL, and 32 mg/dL in the aforementioned groups respectively. Data on lipoprotein (a) levels were not available for the majority of subjects.

## 4 DISCUSSION

Both the 2013 KDIGO and 2018 ACC/AHA cholesterol guidelines recommend at least statin therapy for non‐dialysis dependent CKD patients with ASCVD given that CKD is a major risk factor for progression of ASCVD, particularly with worsening CKD. The ACC/AHA guidelines recommend the use of high‐intensity statins with the addition of ezetimibe and/or PCSK9i specifically to achieve a cardioprotective LDL‐C goal of <70 mg/dL. Based on these guidelines, all patients with CKD in our study should be receiving statin therapy, ideally of high intensity, and many should also be on ezetimibe and/or PCSK9i. However, we found that statin use is not ubiquitous and rates of high‐intensity statin and ezetimibe use remain low in this patient population.

Multiple studies from before the release of these guidelines demonstrate that rates of statin use are low among the CKD population. Examination of statin use in National Health and Nutrition Examination Surveys (NHANES) participants with CKD between 1999 and 2014 revealed that utilization of statins in patients with CKD and ASCVD was higher than in those with ASCVD alone, but overall statin use in the CKD population remained low.^17^ Data from the NHANES participants showed that statin use increased from 17.6% to 35.7% among 7153 American adults with CKD between 1999–2002 and 2011–2014. After multivariate adjustment, CKD status was not associated with statin use (prevalence ratio, 1.01; 95% CI [0.96–1.08]).^17^ The German Chronic Kidney Disease study enrolled 5217 adult patients with moderately severe CKD in Germany shortly before the publication of the 2013 KDIGO guidelines. Of the 4631 patients eligible for statin use per these guidelines, 2473 were receiving statin therapy at the time.^18^ A study of 581 344 United States veterans aged 50 years or older with non‐dialysis dependent CKD Stages 3–5 examined statin use from pharmacy dispensing records between 2012 and 2013. Of patients with CKD overall, 62.1% used statins in 2012. Statin use increased to 76.2% among veterans with both CKD and ASCVD in 2012.^19^

Our study is the first to examine changes in statin use since the publication of the 2018 ACC/AHA guidelines. In an improvement from the aforementioned studies, we found that statin use was near or above 80% at all eGFRs, but there was no significant change in these rates over 2 years. Furthermore, the prevalence of high‐intensity statin use and ezetimibe use remain low across all stages of CKD. Overall, approximately 20% of patients experienced LLT intensification over 2 years. In the PCSK9i cohort, only 12.5% of patients had LLT intensification, most likely because their LDL‐C levels had more often achieved goal. We did not note any significant differences in the use of any LLT between CKD stages.

To further understand patient and clinician perceptions of the role of LLT, a follow‐up study examined the structured questionnaires conducted in the 5006 patients and 113 physician providers of the GOULD registry.^20^ Of all patients, 46% believed that ASCVD was the leading cause of death in women and 63% in men. Less than 30% of patients were able to state that the primary reason for their LLT use was to lower the risk of heart attack or stroke. Additionally, only about 30% knew their LDL‐C levels and the goal. From the physicians' perspective, 66% felt that the biggest barrier to medication adherence was lack of understanding of the importance of statins, compared to costs (9%) or side effects (1%).^20^ While this survey was focused on ASCVD overall and did not further examine views among patients with CKD, patient education about the efficacy and safety of statins is likely still a major contributor to the suboptimal prevalence of statin use among this population. The latter is particularly evident in the rates of statin discontinuation due to concerns about muscle pain. It is also possible that there is not widespread implementation of the KDIGO and ACC/AHA guidelines by physicians due to a lack of awareness or clinical inertia. There may also be some concern for the safety or efficacy of statins in patients with CKD, though multiple studies have demonstrated benefit and safety in this population. These results present an opportunity and a need for increased education of clinicians and patients regarding the role of statins in patients with CKD.

## 5 CONCLUSION

While overall statin use among patients with CKD is high, there have only been modest increases in the use of high‐intensity statin, ezetimibe, and PCSK9i over the course of this prospective 2‐year study. The use of ezetimibe and PCSK9i did not differ by CKD stage, but unfortunately, statin use was lower in those with lower eGFR. Studies of PCSK9i and ezetimibe have shown similar benefits in cardiovascular outcomes across eGFR groups in this high‐risk population.^21^ Despite ASCVD being a major cause of morbidity and mortality in patients with CKD, a majority of patients in this study did not achieve an atheroprotective LDL‐C goal of <70 mg/dL. This highlights a key opportunity to improve quality of care and outcomes in the CKD population.

## STUDY REGISTRATION

ClinicalTrials.gov identifier NCT02993120 (https://clinicaltrials.gov/ct2/show/NCT02993120).

## DISCLOSURES

Dr. Aleesha Shaik reports no relevant disclosures. Dr. Mikhail Kosiborod discloses the following relationships ‐ Research grants from AstraZeneca, Boehringer Ingelheim; other research support from AstraZeneca; consulting honoraria from Applied Therapeutics, AstraZeneca, Boehringer Ingelheim, Novo Nordisk, Sanofi, Amgen, GSK, Merck (Diabetes), Eisai, Intarcia, Novartis, and Glytec. Dr. James de Lemos discloses the following relationships ‐ Consulting income from Amgen, Regeneron, Janssen, Esperion, and Novo Nordisk. Qi Gao reports no relevant disclosures. Dr. Katherine Mues discloses the following relationships ‐ Employed by Amgen. Dr. Shushama Alam discloses the following relationships ‐ Employed by Amgen. Dr. Deepak L. Bhatt discloses the following relationships ‐ Advisory Board: Bayer, Boehringer Ingelheim, Cardax, CellProthera, Cereno Scientific, Elsevier Practice Update Cardiology, Janssen, Level Ex, Medscape Cardiology, Merck, MyoKardia, NirvaMed, Novo Nordisk, PhaseBio, PLx Pharma, Regado Biosciences, Stasys; Board of Directors: AngioWave (stock options), Boston VA Research Institute, DRS.LINQ (stock options), Society of Cardiovascular Patient Care, TobeSoft; Chair: Inaugural Chair, American Heart Association Quality Oversight Committee; Data Monitoring Committees: Acesion Pharma, Assistance Publique‐Hôpitaux de Paris, Baim Institute for Clinical Research (formerly Harvard Clinical Research Institute, for the PORTICO trial, funded by St. Jude Medical, now Abbott), Boston Scientific (Chair, PEITHO trial), Cleveland Clinic (including for the ExCEED trial, funded by Edwards), Contego Medical (Chair, PERFORMANCE 2), Duke Clinical Research Institute, Mayo Clinic, Mount Sinai School of Medicine (for the ENVISAGE trial, funded by Daiichi Sankyo; for the ABILITY‐DM trial, funded by Concept Medical), Novartis, Population Health Research Institute; Rutgers University (for the NIH‐funded MINT Trial); Honoraria: American College of Cardiology (Senior Associate Editor, Clinical Trials and News, ACC.org; Chair, ACC Accreditation Oversight Committee), Arnold and Porter law firm (work related to Sanofi/Bristol‐Myers Squibb clopidogrel litigation), Baim Institute for Clinical Research (formerly Harvard Clinical Research Institute; RE‐DUAL PCI clinical trial steering committee funded by Boehringer Ingelheim; AEGIS‐II executive committee funded by CSL Behring), Belvoir Publications (Editor in Chief, Harvard Heart Letter), Canadian Medical and Surgical Knowledge Translation Research Group (clinical trial steering committees), Cowen and Company, Duke Clinical Research Institute (clinical trial steering committees, including for the PRONOUNCE trial, funded by Ferring Pharmaceuticals), HMP Global (Editor in Chief, Journal of Invasive Cardiology), Journal of the American College of Cardiology (Guest Editor; Associate Editor), K2P (Co‐Chair, interdisciplinary curriculum), Level Ex, Medtelligence/ReachMD (CME steering committees), MJH Life Sciences, Oakstone CME, Piper Sandler, Population Health Research Institute (for the COMPASS operations committee, publications committee, steering committee, and USA national co‐leader, funded by Bayer), Slack Publications (Chief Medical Editor, Cardiology Today's Intervention), Society of Cardiovascular Patient Care (Secretary/Treasurer), WebMD (CME steering committees), Wiley (steering committee); Other: Clinical Cardiology (Deputy Editor), NCDR‐ACTION Registry Steering Committee (Chair), VA CART Research and Publications Committee (Chair); Research Funding: Abbott, Acesion Pharma, Afimmune, Aker Biomarine, Amarin, Amgen, AstraZeneca, Bayer, Beren, Boehringer Ingelheim, Boston Scientific, Bristol‐Myers Squibb, Cardax, CellProthera, Cereno Scientific, Chiesi, CSL Behring, Eisai, Ethicon, Faraday Pharmaceuticals, Ferring Pharmaceuticals, Forest Laboratories, Fractyl, Garmin, HLS Therapeutics, Idorsia, Ironwood, Ischemix, Janssen, Javelin, Lexicon, Lilly, Medtronic, Merck, Moderna, MyoKardia, NirvaMed, Novartis, Novo Nordisk, Owkin, Pfizer, PhaseBio, PLx Pharma, Recardio, Regeneron, Reid Hoffman Foundation, Roche, Sanofi, Stasys, Synaptic, The Medicines Company, 89Bio; Royalties: Elsevier (Editor, Braunwald's Heart Disease); Site Co‐Investigator: Abbott, Biotronik, Boston Scientific, CSI, Endotronix, St. Jude Medical (now Abbott), Philips, Svelte; Trustee: American College of Cardiology; Unfunded Research: FlowCo, Takeda. Dr. Chris Cannon discloses the following relationships ‐ Research grants: Amgen, Boehringer‐Ingelheim (BI), Bristol‐Myers Squibb (BMS), Daiichi Sankyo, Janssen, Merck, Novo Nordisk, Pfizer. Consulting fees: Aegerion, Alnylam, Amarin, Amgen, Applied Therapeutics, Ascendia, BI, BMS, Corvidia, Eli Lilly, HLS Therapeutics, Innovent, Janssen, Kowa, Lexicon, Merck, Pfizer, Rhoshan, Sanofi. Dr. Christie Ballantyne has received grant/research support (to his institution) from Abbott Diagnostic, Akcea, Amgen, Esperion, Ionis, Novartis, Regeneron, and Roche Diagnostic, and has been a consultant for Abbott Diagnostics, Althera, Amarin, Amgen, Arrowhead, AstraZeneca, Corvidia, Denka Seiken, Esperion, Genentech, Gilead, Matinas BioPharma Inc, New Amsterdam, Novartis, Novo Nordisk, Pfizer, Regeneron, Roche Diagnostic, and Sanofi‐Synthelabo. Dr. Robert Rosenson discloses the following relationships ‐ research grants from Akcea, Amgen, AstraZeneca, Medicines Company and Regeneron; consulting honorarium from Akcea, C5, CVS Caremark Corvidia; honoraria from Amgen, Kowa, Pfizer; and Regeneron, royalties from UpToDate, Inc; and stock ownership in MediMergent, LLC.

## Data Availability

Qualified researchers may request data from Amgen clinical studies. Complete details are available at http://www.amgen.com/datasharing.
